# Automated characterization and analysis of expression compatibility between regulatory sequences and metabolic genes in *Escherichia coli*

**DOI:** 10.1016/j.synbio.2024.05.010

**Published:** 2024-05-17

**Authors:** Xiao Wen, Jiawei Lin, Chunhe Yang, Ying Li, Haijiao Cheng, Ye Liu, Yue Zhang, Hongwu Ma, Yufeng Mao, Xiaoping Liao, Meng Wang

**Affiliations:** a School of Life Sciences, Division of Life Sciences and Medicine, University of Science and Technology of China, Hefei, 230026, China; b Tianjin Institute of Industrial Biotechnology, Chinese Academy of Sciences, Tianjin, 300308, China; c Key Laboratory of Engineering Biology for Low-Carbon Manufacturing, Tianjin, 300308, China; d School of Biological Engineering, Tianjin University of Science and Technology, Tianjin, 300457, China

**Keywords:** Regulatory sequences, Protein expression, Fluorescent fusion proteins, Compatibility, Machine learning

## Abstract

Utilizing standardized artificial regulatory sequences to fine-tuning the expression of multiple metabolic pathways/genes is a key strategy in the creation of efficient microbial cell factories. However, when regulatory sequence expression strengths are characterized using only a few reporter genes, they may not be applicable across diverse genes. This introduces great uncertainty into the precise regulation of multiple genes at multiple expression levels. To address this, our study adopted a fluorescent protein fusion strategy for a more accurate assessment of target protein expression levels. We combined 41 commonly-used metabolic genes with 15 regulatory sequences, yielding an expression dataset encompassing 520 unique combinations. This dataset highlighted substantial variation in protein expression level under identical regulatory sequences, with relative expression levels ranging from 2.8 to 176-fold. It also demonstrated that improving the strength of regulatory sequences does not necessarily lead to significant improvements in the expression levels of target proteins. Utilizing this dataset, we have developed various machine learning models and discovered that the integration of promoter regions, ribosome binding sites, and coding sequences significantly improves the accuracy of predicting protein expression levels, with a Spearman correlation coefficient of 0.72, where the promoter sequence exerts a predominant influence. Our study aims not only to provide a detailed guide for fine-tuning gene expression in the metabolic engineering of *Escherichia coli* but also to deepen our understanding of the compatibility issues between regulatory sequences and target genes.

## 1 Introduction

Fine-tuning gene expression is one of the critical strategies for developing efficient microbial cell factories [[1], [2], [3]]. To this end, various standardized libraries of regulatory sequences, including promoter and ribosome binding site (RBS) libraries, have been developed for the precise regulation of genes of interest (GOIs) in different organisms [[4], [5], [6]]. Typically, these regulatory sequences are evaluated through quantitative characterization, employing easily detectable reporter proteins, such as green/red fluorescent protein (GFP/RFP). However, the use of these sequences to regulate the expression of diverse proteins often yields results that greatly differ from expectations. For example, the coefficient of determination (R^2^) for the fluorescence levels of GFP and RFP reporters, controlled by the same set of regulatory sequences (promoter-RBS combinations), is as low as 0.38 [7]. Moreover, using the same promoter-RBS combination, the fluorescence levels of two highly similar RFP variants can vary by as much as 530-fold [8]. These findings suggest that the regulatory sequence intensity determined using a single reporter protein might not be directly applicable to other GOIs.

In recent years, computer-aided design tools and machine learning algorithms have become available for predicting the strength of promoters [[9], [10], [11]] or RBS [8,[12], [13], [14]], such as the well-known RBS Calculator [8]. Nonetheless, these tools often rely on biased datasets characterized by a few reporter proteins that have limitations in genetic diversity [5,7,[15], [16], [17], [18], [19]], or by correlating natural regulatory sequences with protein expression levels of target genes using transcriptomic and proteomic approaches that provide broader gene coverage but with limited exploration of individual gene regulatory sequences [20,21]. Although the above studies offer some reference value in the selection of regulatory sequences, they cannot fully capture the compatibility between regulatory sequences and genes. As a result, these tools may not always accurately predict the expression levels for novel combinations of regulatory sequences and GOIs. Therefore, experimental biologists frequently need to test a broader array of recommended regulatory sequences to identify those that achieve the desired expression levels [22]. This process significantly complicates the task of multi-gene, multi-level combinatorial optimization and can lead to unforeseen or even contradictory results. For instance, genes believed to be downregulated might actually be upregulated. Alternatively, biologists may choose to characterize specialized libraries for individual key genes, typically by creating fusions of the GOI with a fluorescent protein gene [6]. This approach enables the selection of regulatory sequences that guarantee a range of expression levels, but might be too costly when many genes are involved and lacks transferability and general applicability.

Here, to investigate the extent and prevalence of expression compatibility between regulatory sequences and various GOIs, we undertook a systematic study of protein expression levels using multiple regulatory sequences combined with multiple GOIs in the model organism *Escherichia coli*. The protein expression levels were represented by the fluorescence of fusion proteins consisting of the first 180 bp of GOIs with the GFP gene, regulated by different regulatory sequences. These characterization experiments were carried out at the TIB biofoundry [[23], [24], [25]] to rapidly and cost-effectively generate large-scale, high-quality experimental data. A comprehensive fusion protein expression regulation landscape was rapidly established, encompassing core metabolic engineering target genes across multiple levels. This provides a valuable reference for the precise regulation of genes in *E. coli*. Furthermore, we have developed various machine learning models and found that combining regulatory sequence and CDS features enhances protein expression level prediction and achieves improved predictive capability for protein expression levels (Spearman correlation of 0.72). We believe that this extensive dataset of multiple regulatory sequence-GOI combinations will facilitate a deeper understanding of the complex interactions driving gene expression in the future.

## 2 Materials and methods

### 2.1 Bacterial strains and culture conditions

Strains used in this study were listed in Supplementary Table S1. *E. coli* DH5α was used as the host for cloning and plasmid propagation. The plasmids carrying the *ccdB* gene were constructed in the host strain *E. coli* DB3.1. The strain *E. coli* MG1655-WX (MG1655 Δ*ldhA*, Δ*pflB*, Δ*adhE*, *ΔpoxB*, *ΔackA*, *Δpta*) stored in our laboratory was used for the characterization of regulatory sequences and enzyme activity levels. SOC medium (20 g/L tryptone, 5 g/L yeast extract, 10 mM NaCl, 2.5 mM KCl, 10 mM MgCl_2_, 10 mM MgSO_4_, 20 mM glucose) was used to culture strains at 37 °C aerobically, either as liquid culture in 96-well microtiter plates or on 2 % agar plates. To avoid medium background, bacterial cells used for fluorescence determination were grown in M9 minimal medium (6.8 g/L Na_2_PO_4_, 3 g/L KH_2_PO_4_, 0.5 g/L NaCl, 1 g/L NH_4_Cl, 2 mM MgSO_4_, 100 μM CaCl_2_, with 10 g/L glucose) [26]. The cultures were inoculated at a 1 % ratio and grown at 37 °C to an OD_600_ of 0.4–0.6 before fluorescence measurements. All solid and liquid media were supplemented with 100 mg/L ampicillin when cultivating strains carrying pSC101-derived plasmids. Strains were preserved in 500 μL of the culture broth with 15 % glycerol at −80 °C.

### 2.2 Plasmid construction

The pSC101-ccdB-GFP plasmid was constructed as a universal characterization backbone vector for fluorescent protein expression. The *pSC101-AmpR* fragment was amplified using the primer pair 1C2-F/R from the plasmid pSC101-AmpR. Then, the *gfp* gene fragment was amplified from the plasmid pGFP using the primer pair 1C3-F/R, and the *ccdB* gene fragment was amplified from the plasmid pccdB using the primer pair 1C1-F/R. These fragments were assembled into one plasmid through Gibson assembly [27], generating the pSC101-ccdB-GFP plasmid. Subsequently, the negative selection marker *ccdB* of the pSC101-ccdB-GFP plasmid was replaced with different reported regulatory sequences by direct synthesis, generating the GFP expression plasmids. From these, pSC101-derived sfGFP expression plasmids were produced by replacing the *gfp* gene with the *sfgfp* gene under the appropriate regulatory sequences, through direct synthesis. The p15A-derived sfGFP expression plasmid with the regulatory sequence of PR70 was directly fully synthesized, and was used as the backbone for other p15A-derived sfGFP expression plasmids by replacing regulatory sequences through direct synthesis. The pSC101-negative-GFP plasmid without any regulatory sequence was constructed to serve as a negative control in fluorescence assays. The plasmid was amplified into two linear fragments using primers pair 2C2-F/R and 2C3-F/R and then ligated together based on the pSC101-ccdB-GFP.

The fusion protein expression plasmids containing the initial 180 bp from the coding sequences of 37 endogenous and 4 heterologous target genes without codon optimization, alongside 15 artificial and 37 natural regulatory sequences were directly synthesized into the pSC101-ccdB-GFP plasmid by replacing the *ccdB* gene. Synthetic genes were ordered through the GenScript Gene Synthesis service (GenScript, Zhenjiang, Nanjing, China). The plasmids were synthesized in a 96-well source plate to facilitate subsequent robotic workstation operations.

The pSC101-ICDH-320 plasmid was constructed for the expression of ICDH under the control of the regulatory sequence PR320. The sequence of the plasmid backbone containing PR320 was amplified from the plasmid pSC101-ccdB-GFP using the primer pair 1136-320-1F/1R, and the *icd* gene fragment was amplified from the genomic DNA of *E. coli* MG1655 by using the primer pair 1136-320-2F/2R. The two fragments were assembled into one plasmid through Gibson assemble method [27]. The pSC101-derived plasmids for the expression of ICDH and G6PDH under the control of different regulatory sequences were also constructed the same way, using the primer pairs listed in Supplementary Table S2.

All the linearized DNA fragments used in plasmid construction were generated by PCR using the KOD-Plus-Neo kit (TOYOBO, Japan) according to the manufacturer's recommendations. All plasmids were assembled via *in vitro* homologous recombination using the ClonExpress II One Step Cloning Kit (Vazyme, China).

Detailed information on the plasmids and primers used in this study is listed in the Supplementary Table S2.

### 2.3 High-throughput automated characterization of the fluorescence levels produced by diverse combinations of regulatory sequences with target genes

Our integrated robotic system was used to automate the characterization of regulatory sequences, including preparation of competent cells, plasmid transformation, agar plating, colony picking, determination of fluorescence and biomass, etc. A Python data processing script was written to analyze the data files. Firstly, a Beckman i7 96-channel pipettor (Beckman-Biomek, USA) was used to pack the medium stored in the dispensing tank (Beckman) into 96-deep-well plates (Corning, USA) at 800 μL per well and transfer the activated MG1655-WX culture to the plates. The transferred bacterial solution was incubated in a high-throughput incubator shaker (INFORS HT, Switzerland) to an OD_600_ = 0.4–0.6 and immediately placed on the pre-cooled Beckman i7 cryogenic module for 10 min. MG1655-WX competent cells were prepared using an *E. coli* Transformation Kit (TaKaRa, China). The transformation of *E. coli* MG1655-WX with the plasmids was conducted using a published protocol [25]. Briefly, the corresponding plasmids were added to the 96-well PCR plates containing the competent cells, mixed, and then cooled at 4 °C for 10 min, followed by heat shock (90 s at 42 °C). The shocked cells were transferred to 96-deep-well plates with SOC liquid medium and incubated in a shaker (INFORS HT, Switzerland) at 37 °C for 1 h. Following incubation, the cell suspension was centrifuged at 4000 r/min for 2 min, the supernatant was removed using an i7 96-channel pipettor and 100 μL were retained and resuspended. Then, 50 μL of the resuspended solution was plated onto SOC agar plates containing appropriate antibiotics using the QPix 420 clone picker (Molecular Devices, USA) and incubated at 37 °C until colonies appeared. The QPix 420 clone picker was also used to select 3 clones into pre-packaged SOC medium. After overnight culture, 1 % of the resulting seed culture was used to inoculate M9 medium and grown to the logarithmic phase to determine fluorescent protein expression as described above. To determine the fluorescence level, the pipette workstation transferred 150 μL of bacterial suspension into the wells of ELISA plates, and the robotic arm sent them to the SpectraMax iD3 microplate reader (Molecular Devices, America) to simultaneously measure the GFP fluorescence and OD_600_. The output *.csv file was processed using a Python script to obtain the outputs. Then, we performed different operations on abnormal samples according to different output files, including re-measuring after dilution of the bacterial solution, measuring after prolonged culture of the bacterial solution, or re-transformation with the target plasmids. The output files that needed the culture to be re-diluted were automatically re-measured as the input files of the i7 pipette workstation to obtain the correct output files. In case of abnormal fluorescence results, the plasmids in corresponding strains were revalidated by sequencing. Finally, all valid characterization data were recorded for further downstream analysis. All fluorescence values of fusion proteins were uniformly pre-processed by subtracting the fluorescence value of the negative control (the pSC101-negative-GFP construct) to obtain raw data. Log-transformation is typically used to approximate a normal distribution in the correlation analyses and to stabilize variance [28]. Whereas, the fluorescence values of some samples became negative after subtraction of the control. Therefore, a uniform addition of 3000 was applied to all the data before subsequent log_10_-transformation, and used for correlation analysis (Supplementary Table S3). A heat map showing the log_10_-transformated fluorescence levels was constructed using TBtools (https://github.com/CJ-Chen/TBtools) [29].

### 2.4 High-throughput automated RNA extraction

To measure the relative abundance of mRNAs, the RNA extraction was done using the Kingfisher automated extraction instrument based on magnetic particle processing (Thermo Fisher, America) on the basis of bacterial cells pre-treated with the RNAprep Pure Bacteria Kit (Tiangen, China). RNA enzymes and nucleic acid scavengers (Vazyme, China) were used to eliminate RNase and DNase interference. Agarose gel electrophoresis and photometry were used to determine the quality of the extracted RNA samples.

### 2.5 RT-qPCR analysis

The cDNA was obtained from the reverse-transcription of total mRNA using the ReverTra Ace qPCR RT Master Mix kit (Toyobo, Japan). The cDNA samples were stored at −20 °C for subsequent qPCR and analysis.

The analysis was conducted on a LightCycler 480 Real-time PCR System (Roche, Germany) using the following parameters: 95 °C for 20 s; 40 cycles: 95 °C for 1 s, 60 °C for 20 s, 72 °C for 20 s. The primers are listed in Supplementary Table S2. The delta-delta cycle threshold (ΔΔCt) method was used to calculate the relative mRNA levels of the target genes.

### 2.6 Automated preparation of crude lysates and measurement of enzyme activity

Cells of *E. coli* MG1655-WX containing ICDH or G6PDH expression plasmids were cultured to the logarithmic phase for enzyme activity characterization. Three biological replicates of all samples were cultured in 96-well plates, harvested by centrifugation and washed with PBS. After another round of centrifugation, the PBS supernatant was removed and the lysis buffer (1 %, with PMSF) was used to re-suspend the cell pellet, after which the cells were lysed by repeatedly freezing at −80 °C and thawing at 4 °C. The lysate was centrifuged at 4 °C, and the supernatant was collected to determine the enzyme activity. The concentrations of the crude enzymes were measured using the Micro BCA Protein Assay Kit (CoWin Biotech, China).

ICDH and G6PDH enzyme activities were measured using commercial kits (Solarbio, China) by monitoring NADPH formation at 340 nm. All operations were performed on the automated integrated system. The Beckman i7 96-channel pipettor and SpectraMax iD3 microplate reader (Molecular Devices, USA) were used to complete the multi-step experimental operations. One unit of ICDH/G6PDH activity was defined as the amount of enzyme required to reduce 1 μmol of NADP^+^ to NADPH in 1 min.

### 2.7 Machine learning models for predicting the protein expression levels of fusion proteins

To investigate the influence of regulatory sequences and fusion protein coding sequences on fusion protein fluorescence values, we employed an end-to-end automated machine-learning approach using BioAutoMATED [30]. Based on the log_10_-transformated fluorescence values (Supplementary Table S3), machine learning models were constructed for sequence data (comprising promoter, RBS, 180 bp of the gene, and (GGGGS)_3_ linker alongside the GFP gene) and preprocessed fluorescence values using TOPT [31], DeepSwarm [32] and AutoKeras [33] architectures, with 85 % of the dataset allocated for training and 15 % for validation.

In addition, traditional methods based on one-hot encoding and k-mer techniques (k = 6) were applied for sequence encoding. XGBoost [34] and GBDT [35] models were then employed for prediction using the encoded dataset. To evaluate the performance of different methods, we utilized the sklearn library [36], specifically its metrics module, which includes functions for calculating the R^2^ score, Pearson correlation coefficient, and Spearman correlation coefficient. These metrics allowed us to measure the correlation between predicted and experimental values, helping us to compare the effectiveness of various methods.

### 2.8 Ablation studies to assess the individual impact of different components

Firstly, each component (promoter, RBS or CDS) was considered individually. Individual models were trained using datasets where only one component varied while the others were held constant. For instance, to scrutinize the influence of the promoter, models were trained utilizing datasets featuring diverse promoters while maintaining the RBS and CDS constants. Following this, the prediction results from all the individual models were integrated to encompass various combinations of RBS and CDS. This integration allowed for a comprehensive evaluation against the true values. The same approach was applied to assess the impact of RBS and CDS individually.

Furthermore, combinations of factors were considered. Models were trained using data points where combinations of two factors varied while the third factor remained constant. For instance, models were trained using data points with varying combinations of promoter and RBS while keeping the CDS constant. Similarly, combinations of promoter and CDS, as well as RBS and CDS, were evaluated. The performance of each model was evaluated using the R^2^ score, Pearson correlation coefficient, and Spearman correlation coefficient. Comparisons were made to determine the relative impact of different factors on protein expression levels.

## 3 Results

### 3.1 Changing experimental conditions can lead to significant differences in fluorescence levels

**Fig. 1:**
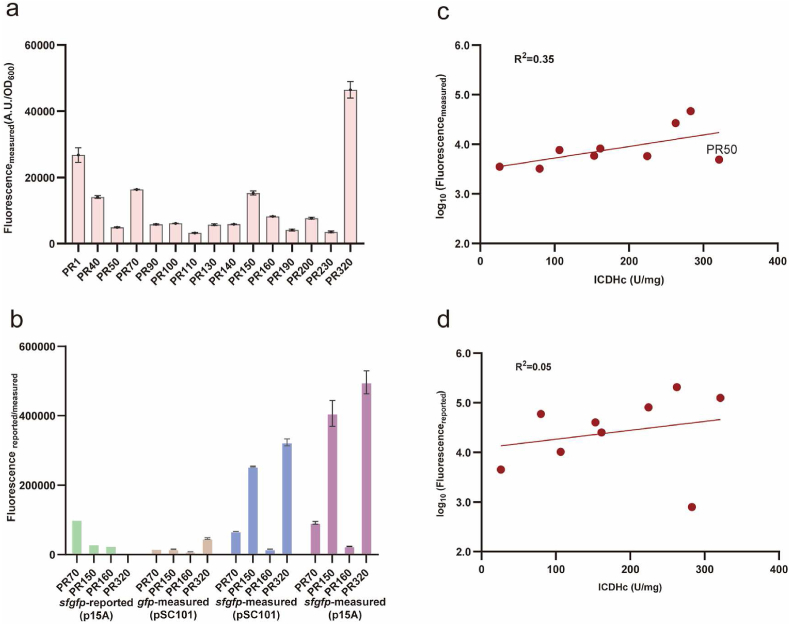
Incompatibility of protein expression levels between regulatory sequences and target genes. **a**, Measured fluorescence values using the *gfp* gene from our laboratory under the control of 15 regulatory sequences. **b,** Comparison of fluorescence values based on different replicons and fluorescent proteins from the reported or measured data in this study. Four regulatory sequences (PR70, PR150, PR160 and PR320) were compared. Correlation analysis of measured fluorescence values (**c**) and reported fluorescence values (**d**) with ICDH enzyme activities under the control of the same regulatory sequences (PR1, PR50, PR90, PR110, PR140, PR160, PR200, PR230 and PR320).

To evaluate if the fluorescence intensities reported for an artificial library can serve as a reliable reference, we chose 15 candidate regulatory sequences from a prominent promoter-RBS combination library [5] with varying reported fluorescence intensities (ranging from ∼200,000 to ∼800, subsequently renamed PR1-PR320 based on their reported strength; see Table 1 and Supplementary Fig. 1a). Initially, we constructed 15 GFP expression vectors derived from stable and low-copy pSC101, utilizing the GFP coding gene *gfp* available in our laboratory. A significant discrepancy in the strength rankings of the regulatory sequences was observed when comparing the results of this study to those previously reported in the literature (Fig. 1a and Supplementary Fig. 1a).

**Table 1:** Strength reassessment of 15 groups of candidate regulatory sequences from the literature [5].

ID	Promoter--RBS	Fluorescence-reported	Renamed as	Fluorescence-measured
PR1	BBa_J23119--apFAB917	205925.43	RS2	26764.64
PR40	apFAB57--apFAB904	151203.55	RS5	14069.18
PR50	BBa_J23119--BBa_J61129	125599.20	RS12	4934.48
PR70	apFAB46--BBa_J61113	100184.78	RS3	16366.07
PR90	apFAB70--BBa_J61127	80517.42	RS10	5791.25
PR100	BBa_J23118--apFAB923	71086.95	RS8	6101.91
PR110	BBa_J23109--apFAB917	59593.91	RS15	3218.75
PR130	apFAB47--B0032_RBS	50356.96	RS11	5714.72
PR140	apFAB55--apFAB876	40225.67	RS9	5866.55
PR150	pTrc*--apFAB912	30006.37	RS4	15268.69
PR160	BBa_J23100--apFAB918	25164.19	RS6	8228.28
PR190	apFAB277--apFAB876	10857.19	RS13	4109.56
PR200	BBa_J23105--apFAB857	10232.15	RS7	7686.22
PR230	BBa_J23115--apFAB904	4502.01	RS14	3537.41
PR320	apFAB75--JBEI_RBS	793.90	RS1	46446.60

We suspected that differences between the GFP expression vectors (p15A versus pSC101 replicon), culture conditions (LB versus M9 medium), and measurement methods (flow cytometry versus microplate reader) might have contributed to inconsistencies in the evaluation results compared to the literature. To further investigate this discrepancy, we also created sfGFP expression vectors (four derived from pSC101 and four from p15A) using the same *sfgfp* gene sequence cited in the literature [5], which shares 95.04 % amino acid sequence similarity with the *gfp* used in this study (Supplementary Fig. 1b). The results demonstrated that, even when employing the identical sfGFP sequence, the strength ranking of regulatory sequences diverged from the literature-reported results (*sfgfp*-reported versus *sfgfp*-measured, Fig. 1b). This indicated that the fluorescence intensities from an artificial library cannot be straightforwardly used as references without re-characterization under specific culture conditions. Moreover, under identical conditions, significant differences were noted in the regulatory sequence strength rankings when paired with different GFP target genes (*gfp* versus *sfgfp*, Fig. 1b), suggesting the necessity for re-characterization across various target genes.

### 3.2 Fluorescence levels cannot accurately reflect the specific enzyme activity of metabolic genes under the control of same regulatory sequences

In addition to the fluorescent protein gene, we evaluated the expression compatibility of regulatory sequences using actual metabolic genes. Specific enzyme activity assays were performed to gauge the expression levels of the corresponding proteins. Accordingly, we constructed pSC101-derived expression vectors for isocitrate dehydrogenase (ICDH, encoded by the *icd* gene) using various regulatory sequences, including PR1, 50, 90, 110, 140, 160, 200, 230, 320. As depicted in Fig. 1c and d, we identified only weak correlations between the specific enzyme activities of ICDH and the corresponding fluorescence intensities measured in this study (R^2^ = 0.35) or those reported in the literature (R^2^ = 0.05). Intriguingly, PR50, despite its low measured fluorescence intensity (Fig. 1a), resulted in the highest specific enzyme activity (Fig. 1c). However, when compared to other regulatory sequences whose fluorescence intensities were within a 2-fold difference from PR50, the specific enzyme activities displayed substantial variation, resulting in a maximal 11-fold discrepancy (ranging from 321.2 to 26.0 U/mg, Fig. 1c). Additionally, specific enzyme activity assays for glucose-6-phosphate dehydrogenase (G6PDH, encoded by *zwf*) exhibited similarly weak correlations (R^2^ = 0.10 with measured fluorescence intensity, Supplementary Fig. 1c). These findings further substantiate that neither the reported nor the re-measured fluorescence intensities of regulatory sequences can be directly applied to the precise regulation of GOIs.

### 3.3 Fusion fluorescent proteins can better reflect the specific enzyme activity of metabolic genes

**Fig. 2:**
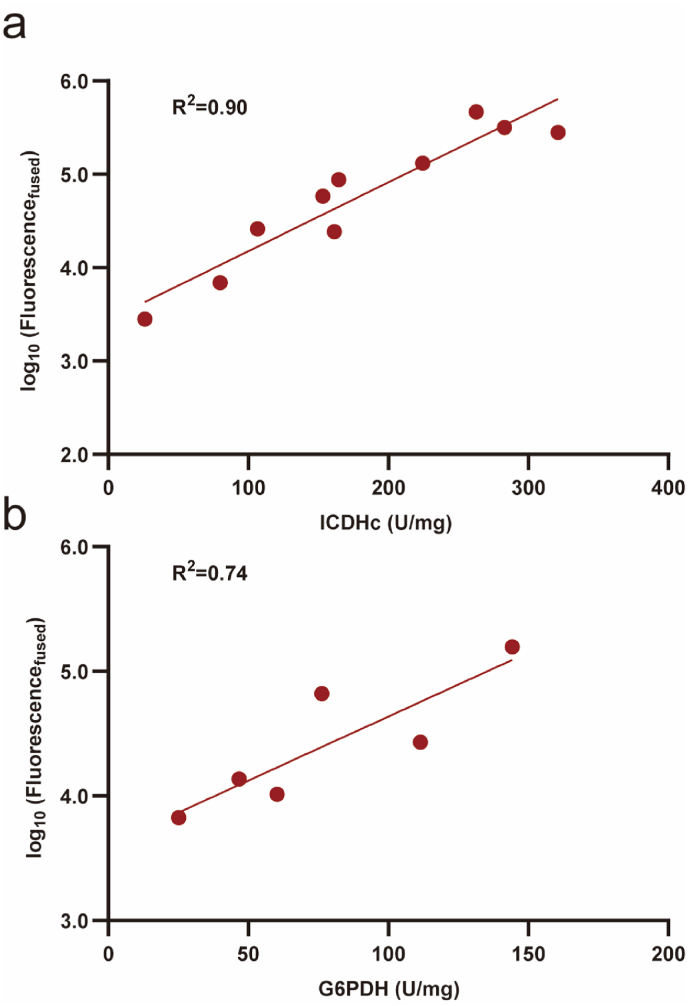
Correlation analysis of measured fluorescence values of GFP fusions with enzyme activities. **a**, Correlation analysis of measured fluorescence values of GFP fusions with ICDH enzyme activities under the control of the regulatory sequences PR1, PR50, PR90, PR110, PR140, PR160, PR200, PR230 and PR320. **b**, Correlation analysis of measured fluorescence values of GFP fusions with G6PDH enzyme activities under the control of the regulatory sequences PR1, PR50, PR90, PR100, PR140 and PR160.

Due to the challenge of directly measuring intracellular expression levels of target proteins, many studies fused the coding sequence (CDS) of the target gene with a fluorescent protein CDS [6,22,37]. This approach allows the fluorescence values of the fusion protein to serve as an indirect reflection of the target protein's expression levels. However, this method faces drawbacks, such as the high construction cost associated with fusing the complete gene to a fluorescent protein sequence due to varying gene lengths, and the increased risk of failed fusion protein expression. Moreover, the initial sequence close to the start of the CDS has been confirmed with a vital role in gene expression [16,18], which prompted the development of a strategy that fuses the first 180 bp of the target gene with a flexible linker (GGGGS)_3_ and a fluorescent protein gene [38]. This method ensures that the fusion of regulatory sequences with the first 180 bp of GOI remains under 300 bp, significantly reducing gene synthesis costs. Moreover, the inclusion of a flexible linker minimizes the risk of misfolding. This technique has proven effective in screening and characterizing promoter and/or RBS sequences for various target genes [[38], [39], [40]]. To determine whether this fusion protein strategy could be used to more accurately characterize the expression intensities of various regulatory sequence and metabolic gene combinations, we constructed pSC101-derived expression vectors for *icd*_*180*_*-gfp* and *zwf*_*180*_*-gfp* fusion proteins under the control of different regulatory sequences. The fluorescence intensity of these fusions showed significantly improved correlations with the specific enzyme activities of ICDH (R^2^ = 0.88, Fig. 2a) and G6PDH (R^2^ = 0.74, Fig. 2b), demonstrating that the fusion protein strategy offers a more reliable approach for evaluating the expression of GOIs.

### 3.4 Large-scale fluorescence characterization of fusion proteins underscores the necessity for re-characterizing GOIs with regulatory sequences

To systematically examine the compatibility of regulatory sequences with target genes, we broadened our study to include the combination of 15 regulatory sequences (renamed RS1-RS15 according to their corresponding measured fluorescence strengths using the *gfp* gene, Table 1) and 41 target metabolic genes (Table 2). This gene set encompassed 37 key metabolic genes from *E. coli* [41] and 4 metabolic genes from other species. The combination cassettes (∼260 bp) containing the selected regulatory sequences combined with the first 180 bp of the target genes were directly synthesized into the vector (Supplementary Fig. 2a). As a result, 520 representative plasmids were successfully obtained, while 95 plasmids failed to assemble. Additionally, for endogenous genes from *E. coli* with their wild-type regulatory sequences were also fused with the fluorescent marker to successfully construct 36 plasmids, except for gene *b3236.* These failure to obtain these constructs might be attributed to the potential metabolic burden [42] induced by these sequence combinations.

**Table 2:** Information on key endogenous and heterologous genes related to the central metabolism of *E. coli*.

Gene_ID	Gene_Name	Encoded enzyme
*b0008*	*talB*	Transaldolase
*b0114*	*aceE*	Pyruvate dehydrogenase E1 component
*b0420*	*dxs*	1-Deoxy-d-xylulose-5-phosphate synthase
*b0720*	*gltA*	Citrate synthase
*b0754*	*aroG*	3-Deoxy-7-phosphoheptulonate synthase
*b1136*	*icd*	Isocitrate dehydrogenase
*b1207*	*prs*	Ribose-phosphate diphosphokinase
*b1603*	*pntA*	Proton-translocating NAD(P) + transhydrogenase subunit alpha
*b1676*	*pykF*	Pyruvate kinase
*b1702*	*ppsA*	Phosphoenolpyruvate synthase
*b1761*	*gdhA*	Glutamate dehydrogenase
*b1852*	*zwf*	Glucose-6-phosphate 1-dehydrogenase
*b1854*	*pykA*	Pyruvate kinase
*b2029*	*gnd*	6-Phosphogluconate dehydrogenase
*b2296*	*ackA*	Acetate kinase
*b2388*	*glk*	Glucokinase
*b2913*	*serA*	Phosphoglycerate dehydrogenase
*b2914*	*rpiA*	Ribose-5-phosphate isomerase A
*b2935*	*tktA*	Transketolase
*b2943*	*galP*	Galactose/H+ symporter
*b3041*	*ribB*	3,4-Dihydroxy-2-butanone-4-phosphate synthase
*b3916*	*pfkA*	6-Phosphofructokinase 1
*b3919*	*tpiA*	Triose-phosphate isomerase
*b4025*	*pgi*	Glucose-6-phosphate isomerase
*b3956*	*ppc*	Phosphoenolpyruvate carboxylase
*b4090*	*rpiB*	Ribose-5-phosphate isomerase
*b3386*	*rpe*	Ribulose 5-phosphate 3-epimerase
*b3403*	*pck*	Phosphoenolpyruvate carboxykinase
*b3612*	*gpmM*	Phosphoglycerate mutase
*b3952*	*pflC*	Pyruvate formate lyase
*b3951*	*pflD*	Pyruvate formate lyase
*b3236*	*mdh*	Malate dehydrogenase
*b4014*	*aceB*	Malate synthase
*b3870*	*glnA*	Glutamine synthetase
*b1779*	*gapA*	NADH hydratase
*b1612*	*fumA*	Fumarase A
*b4232*	*fbp*	Fructose-bisphosphatase
*BSU36010*	*alsS*	Acetolactate synthase from *Bacillus subtilis*
cg1643*	*gnd*	6-Phosphogluconate dehydrogenase mutant S361F from *Corynebacterium glutamicum*
cg1778*	*zwf*	Glucose-6-phosphate 1-dehydrogenase mutant A243T from *Corynebacterium glutamicum*
*CA_C2873*	*acat*	Acetyl-CoA acetyltransferase from *Clostridium acetobutylicum*

The manual characterization of numerous fluorescent fusion proteins is both labor-intensive and error-prone. To address this, we implemented an automated experimental protocol along with automated data filtering and analysis methods for more efficient characterization. We crafted a standardized workflow (Supplementary Fig. 2b) tailored for the TIB biofoundry, facilitating regulatory sequence characterization in *E. coli*. Notably, automated analysis and redesign of fluorescence measurement experiments was included. For example, if a culture fails to reach the necessary density (e.g., OD_600_ < 0.25) or if the fluorescence reading is outside the instrument's optimal range, an abnormal sample report is created. This report triggers the generation of a strain rearrangement file for the liquid handler (i7) in CSV format. Through several iterations of screening, we successfully gathered high-quality characterization data for all constructs.

**Fig. 3:**
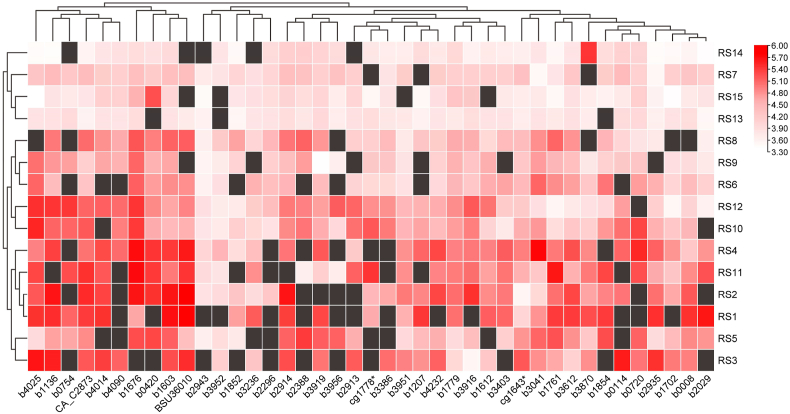
Heat-maps showing the log_10_-transformated fluorescence levels of different combinations of regulatory sequences and metabolic genes. Information corresponding to unsuccessfully constructed samples was marked with black squares. All data were log_10_ transformed. The scale of relative fluorescence levels is displayed on the right. Cluster analysis was conducted based on columns and rows, and the clustering results are displayed on the left and top.

**Fig. 4:**
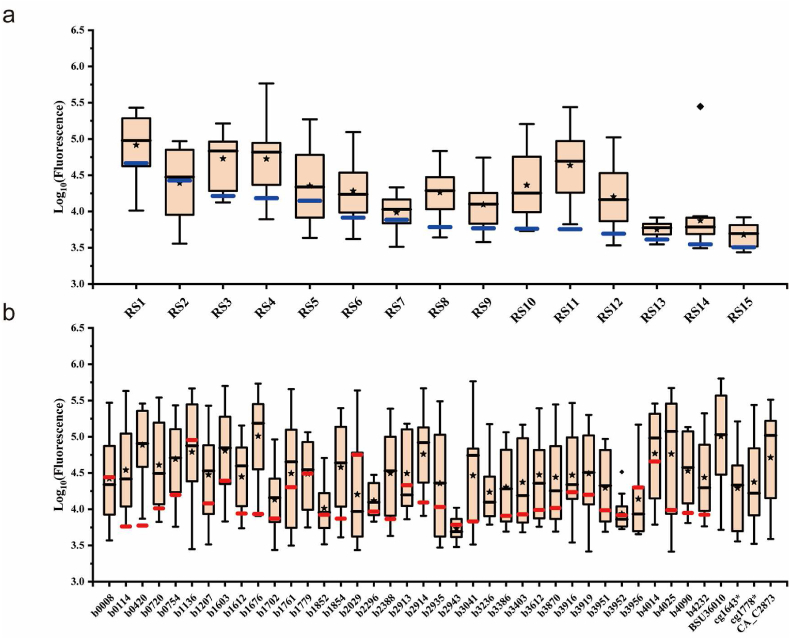
Box-plot showing the distribution of log_10_-transformated fluorescence levels grouped by regulatory sequences (**a**) and metabolic genes (**b**). It displays the minimum (lower bars), median (black horizontal lines), mean (black stars), maximum (upper bars) and outliers (black squares). The orange box represents the data point between first quartile and third quartile. The solid blue line represents the fluorescence intensity of GFP under 15 regulatory sequences, and the red solid line indicates the fluorescence intensity of the fused protein under the control of its corresponding wild-type regulatory sequence.

The GFP fusion protein expression data were log10 transformed as displayed in Fig. 3, Fig. 4. Most of regulatory sequences showed a broad intensity range for different genes (Fig. 4a), with exceptions such as RS13, RS14, and RS15. Regulatory sequences like RS1, RS2, RS3, and RS4, known for their stronger *gfp*-based fluorescence intensity, induce higher expression levels of most fusion proteins. Sequences like RS13, RS14, and RS15, with lower *gfp*-based fluorescence, tended to induce lower expression of most fusion proteins. Nonetheless, a lot of exceptions were noted. For instance, despite the regulatory sequence RS11 demonstrating relatively low fluorescence intensity, many corresponding fusion constructs exhibited high fluorescence intensities. Moreover, despite the regulatory sequence RS2 demonstrating strong expression intensity for the majority of fusion proteins, its log_10_-transformed fluorescence intensity for the fusion with cg1643* was only 3.56, slightly above the lowest observed value of 3.41 across all combinations. Conversely, RS14, which typically showed lower expression intensity for most fusion proteins, exhibited the highest expression intensity for the fusion with *b3870* among the 15 regulatory sequences (Fig. 3). These results further confirm the necessity of re-characterizing the combinations of GOIs with regulatory sequences.

From the perspective of target genes (Fig. 4b), most exhibited a wide range of fluorescence intensities with different regulatory sequences. However, the fluorescence levels of genes *b2943* (encoding a galactose:H^+^ symporter) and *b3952* (encoding a pyruvate formate lyase activating enzyme) remained relatively low. This may be due to these genes being subject to relatively strict regulation. For example, *b2943* has been reported to be under the control of the negative regulation by transcription factors GalS and GalR, with their DNA binding sites being located near the 106 bp mark from the start of the CDS [43]. Interestingly, distinct clustering patterns of genes across the 15 regulatory elements were observed (Fig. 3), which could not be explained by the phylogenetic tree of the 180 bp nucleotide sequence (Supplementary Fig. 3). This illustrates the interaction complexity of regulatory sequences with natural CDS. Notably, the fluorescence intensity of many endogenous genes under the regulation of their natural regulatory sequences (Supplementary Fig. 4) was lower than that under the control of any of the 15 artificial regulatory sequences, such as *b0114*, *b0420* and *b1676* (Fig. 4b). This suggests that selecting specific sequences to downregulate these genes based solely on reported or measured fluorescence intensity (Table 1) rather than re-characterization, might inadvertently result in their upregulation. This may cause significant misinterpretations in subsequent analyses of genotype-phenotype relationships. Therefore, our results also provide a precise reference guide for the regulated expression of these genes in *Escherichia coli* (Supplementary Table S3).

### 3.5 Machine learning based on the combined features of regulatory sequences and CDS enhances the prediction of protein expression levels

**Fig. 5:**
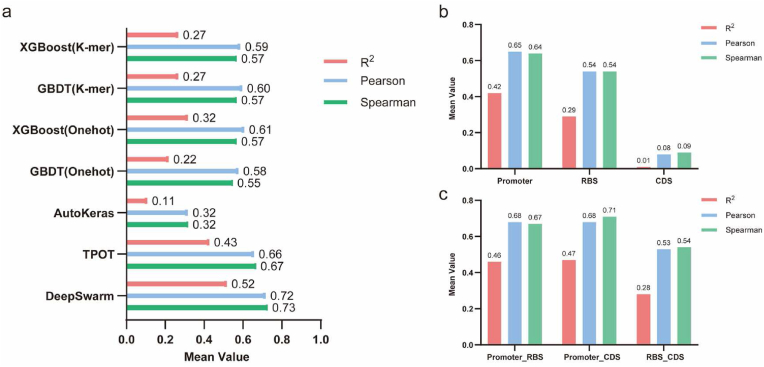
Prediction of fluorescence values of different combinations of promoter, RBS and CDS based on machine learning models. **a**, Comparison of prediction results based on different models for the same dataset. **b,** Ablation study to investigate the effect of the single factors. **c,** Ablation study to investigate the effect of the combinations of factors.

To advance our comprehension of gene expression regulation, we investigated the capability of machine learning models to predict fluorescence intensity resulting from the combination of regulatory and coding sequences. We employed various machine-learning approaches on our collected data. Initially, sequences from 520 valid data points (Fig. 3) were encoded using one-hot and k-mer encoding. Then, we applied XGBoost and GBDT models for prediction, achieving the highest performance with an R^2^ value of 0.32 (Fig. 5a). Furthermore, using the BioAutoMATED tool [30], we constructed automated machine-learning models based on the same dataset. There was a notable improvement in the performance with both DeepSwarm and TOPT architectures. Notably, the DeepSwarm model yielded the best performance, with an R^2^ of 0.52, a Pearson correlation of 0.72, and a Spearman correlation of 0.73 (Fig. 5a).

Furthermore, to systematically assess the influence of different components (promoters, RBS, coding sequences) on final protein expression levels, we conducted further ablation studies. These studies revealed that when analyzing single factors individually, predictions based on the promoter performed the best, followed by those based on RBS, while predictions based solely on the CDS were virtually ineffective (Fig. 5b). This suggests that the promoter has the most substantial impact on the final protein expression level. Moreover, protein expression levels cannot be estimated without considering regulatory sequences, which is consistent with the significant variability we noted in the expression levels of almost all of the 41 tested metabolic genes across the diverse regulatory sequences (Fig. 4b).

Moreover, when evaluating combinations of factors, we observed that the predictive performance was enhanced when the promoter and RBS were considered together, raising the R^2^ to 0.46 (Fig. 5c). This result is closely matched with the fitting outcomes derived from our re-measured GFP data and the average fusion fluorescence data across 41 genes with these regulatory sequences (Supplementary Fig. 5). Although fluorescence intensity data obtained using a single reporter gene cannot precisely guide the regulation of gene expression, they still offer a basic reference guide. Interestingly, we discovered a synergistic effect on the predictive performance when the CDS was paired with the promoter (R^2^ = 0.47), slightly exceeding the combination of promoter and RBS. However, combining CDS with RBS did not further enhance the predictive performance.

### 3.6 Correlation analysis of mRNA and fluorescence levels suggests that the transcription strength as a key determinant of final protein expression levels

**Fig. 6:**
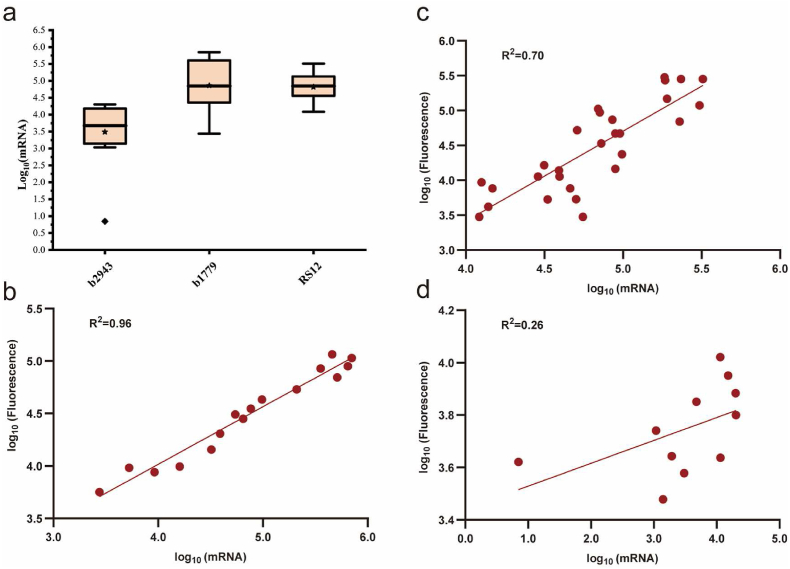
Correlation analysis between transcription and translation levels of fused genes. **a,** The box plot displays the distribution of log_10_-transformated mRNA levels for genes *b2943* and *b1779* or regulatory sequence RS12. **b,** Correlation analysis between the log_10_-transformated mRNA values and log_10_-transformated fluorescence values of the GFP fusions for the same gene (*b1779*). **c,** Correlation analysis between the log_10_-transformated mRNA values and log_10_-transformated fluorescence values of the GFP fusions for different genes under the control of the same regulatory sequence (RS12). **d,** Correlation analysis between the log_10_-transformated mRNA values and log_10_-transformated fluorescence values of the GFP fusions for the same gene (*b2943*).

In light of the fluorescence data set for fusion proteins and results from machine learning analysis, we deemed it necessary to further investigate the relationship between transcription levels and final protein expression levels. To this end, we utilized quantitative real-time PCR to measure the mRNA levels of various gene-regulatory sequence combinations, and subjected these levels to log_10_ transformation for analysis. Among the 41 genes, *b1779* and *b2943* were chosen as representative cases, as the former showed a significant fluorescence intensity gradient under the control different regulatory sequences, whereas the latter did not (Fig. 4b). Our findings revealed that the overall RNA level of *b2943* was markedly lower than that of *b1779*, with the highest log_10_-transformed mRNA level of the former reaching only 4.31, substantially below the latter's average value of 4.86 (Fig. 6a). The overall correlation between transcription and translation levels of the *b1779* gene was notably high, reaching an R^2^ value of 0.96 (Fig. 6b). Furthermore, we examined 28 combinations incorporating the regulatory sequence RS12, but the overall correlation between transcription and translation levels reached a moderate value of R^2^ = 0.70 (Fig. 6c). These findings suggest that the transcription level is the main factor determining the protein expression level, consistent with a recent report [20]. Notably, regulatory sequence RS12 yielded a dramatic range of mRNA levels with a difference of up to a 26-fold across different target genes, leading to a variation of up to 684-fold in final protein expression levels (Fig. 6c). This illustrates that regulatory sequences and CDS jointly affect gene expression, generating a broad spectrum of protein expression levels, in line with a prior study [7]. Furthermore, for gene *b2943*, which exhibited a generally lower transcription level, the mRNA expression level showed a surprisingly weak correlation (R^2^ = 0.26) with protein expression level (Fig. 6d). This observation deviates from the intuitive expectation that lower transcription levels would result in protein expression levels being more heavily influenced by transcriptional changes. This implies that there might be complex translational regulation of the membrane protein gene *b2943* in *Escherichia coli*.

## 4 Discussion

The fine-tuning of gene expression requires accurate prediction of the expression levels of GOIs when combined with standardized regulatory sequences. However, our work confirmed that data on the expression strength of regulatory sequences characterized using a single reporter protein cannot be directly applied to precisely guide the expression of various target genes. Due to differences of experimental condition, the fluorescence intensity rankings can show significant variation even when using the same sfGFP reporter sequence (Fig. 1b). Moreover, under the same experimental conditions, the performance of the same set of regulatory sequences varied greatly with different coding sequences, often failing to correlate well with the recharacterization results obtained using GFP (Fig. 1c and Supplementary Fig. 1c). This is consistent with reports indicating that protein expression levels can significantly change when altering the reporter gene coding sequence [[15], [16], [17]] or comparing different genes [7,18,44]. This makes the optimization work for multiple genes at multiple levels more challenging, necessitating characterization of GOIs with regulatory sequences. To address this, we adopted a fluorescent protein fusion strategy that can reflect the target gene expression strength relatively accurately (Fig. 2), and combined 41 metabolic genes with 15 regulatory sequences (Supplementary Fig. 2a), obtaining fusion fluorescence data for 520 combinations of regulatory sequences with metabolic genes (Fig. 3). The data indicate that for most regulatory sequences, the variation in protein expression levels when regulating different target genes can be substantial, with differences reaching beyond two orders of magnitude in some cases (e.g., RS2, RS3, RS11 and RS14, Fig. 4a). The underlying reasons for this phenomenon are likely complex. For example, the folding stability of mRNA sequences [17,45] or the efficiency of translation initiation [46] and early elongation [16] generated by various combinations can differ significantly. Moreover, issues such as ribosome collision [47] and cellular metabolic regulation [43] may also contribute to these variations. Moreover, it is worth noting that the general fluorescence levels of genes *b2943* and *b3952* remained relatively low (Fig. 4b). Through literature review and database searches, we discovered that *b2943* is reported to be under negative regulation by the transcription factors GalS and GalR, with their DNA-binding sites located near the 106 bp mark from the start of the CDS [43]. This suggests that the method of fusing target gene coding sequences with fluorescent protein sequences can be instrumental in uncovering potential regulatory relationships. This has significant implications for guiding subsequent metabolic engineering efforts aimed at modulating the expression levels of these target genes.

In current research on the expression compatibility of regulatory and coding sequences in prokaryotes, common combinations include mutation libraries of a cassette comprising a single regulatory sequence with a single fluorescent reporter gene [[15], [16], [17]], a single regulatory sequence with multiple metabolic genes [44], multiple regulatory sequences with one or a few fluorescent reporter genes [5,7,18,19], and combinations of natural regulatory sequences with regulated genes based on transcriptomics and proteomics [20,21]. In contrast to these combinations, our study selected artificial regulatory sequences with a range of expression strengths from prominent synthetic libraries and constructed comprehensive combinations with several target genes commonly targeted in metabolic engineering within the *Escherichia coli* chassis (Table 1, Table 2). This dataset is more directly applicable to the development of industrial microbial strains. Therefore, coupled with the fusion fluorescence intensity data for diverse target genes under their inherent regulatory sequences (Supplementary Fig. 4), we offer a predictive landscape for gene expression optimization in the *Escherichia coli* chassis (Fig. 4b), offering biologists a more precise reference for selecting appropriate regulatory sequences. The utilization of a pre-assessment method for protein expression levels of GOI under specific regulatory sequences enables biologists to achieve more precise and purposeful regulation of complex metabolic networks. This approach not only helps avoid the combinatorial explosion associated with multi-gene, multi-level regulation but also prevents misinterpretations in subsequent analyses of genotype-phenotype relationships. While this method still comes with some limitations: (i) Due to the complex interactions among genes, characterization results for individual genes may not accurately reflect the actual expression levels of various proteins under simultaneous multi-gene regulation. (ii) Inability to eliminate the risk of potential misfolding in fusion proteins.

In addition, these combinations of multiple regulatory sequences and genes also offer new perspectives for further studying the compatibility issues between regulatory sequences and target genes. For instance, by combining fluorescence data with machine learning ablation analysis, we found that the main single factor affecting the final protein expression level is the promoter activity (Fig. 5, Fig. 6), aligning with findings from previous research [20]. When promoters are analyzed in conjunction with CDS or RBS, the predictive accuracy for protein expression levels is significantly improved. Moreover, if we truncate the 180 bp to only the first 36 bp in machine learning analysis, the prediction accuracy of the model does not significantly decline (Supplementary Fig. 6). This indicates that the regions near the 5′ end of the CDS have a more substantial impact on the final expression level of the protein, in agreement with previous studies [16,18]. However, in practical experiments, retaining only these 36 bp might overlook potential endogenous regulatory metabolism (e.g., in the case of *b2943*, Fig. 4b), failing to effectively reflect the expression level of the target gene. Nonetheless, given the current dataset's limited size of several hundred data points, achieving high R^2^ predictive accuracy remains a challenge. Future research will necessitate an expansion of the data volume and diversity to enhance predictive precision.

Besides volume and diversity, the reliability and reproducibility of data are also crucial for ensuring the reliability and universality of the final results obtained from machine learning models. However, our research demonstrates that fluorescence intensity rankings can exhibit significant variation even when utilizing the same sfGFP reporter sequence (Fig. 1b). Moreover, manual operations are susceptible to human errors, especially when multiple iterations are necessary, thereby compromising experimental consistency and objectivity. Consequently, in this study, we have established an automated workflow for characterizing fluorescence intensity across various combinations of regulatory sequences and targeted genes (Supplementary Fig. 2b), leveraging the capabilities of the TIB biofoundry. Through this approach, manual experimental tasks have been transitioned to robotic automation, and an in-time analysis of fluorescence data was enabled to evaluate data quality and determine the necessity for subsequent cultivation or dilution prior to fluorescence measurements.

## Code availability

The python implementation is available at https://github.com/Suchcools/RSSP-AutoML.

## CRediT authorship contribution statement

**Wen Xiao:** performed the wet lab experiments, perform the formal analysis, wrote and revised the manuscript, conceived the concept. **Jiawei Lin:** built the machine learning models. **Chunhe Yang:** wrote the characterization, Formal analysis, codes. **Add Ying Li:** built the machine learning models. **Haijiao Cheng:** performed the wet lab experiments. **Ye Liu:** revised the manuscript. **Yue Zhang:** revised the manuscript. **Hongwu Ma:** revised the manuscript, supervised the project. **Yufeng Mao:** performed the, Formal analysis, wrote and revised the manuscript, conceived the concept. **Xiaoping Liao:** performed the, Formal analysis, built the machine learning models, wrote and revised the manuscript. **Meng Wang:** revised the manuscript, conceived the concept, supervised the project. All authors discussed the results and commented on the manuscript.

## Declaration of competing interest

The authors declare that they have no known competing financial interests or personal relationships that could have appeared to influence the work reported in this paper.

## References

[1] Li N., Zeng W., Xu S., Zhou J. (2020). Toward fine-tuned metabolic networks in industrial microorganisms. Synthetic and Systems Biotechnology.

[2] Li C., Jiang T., Li M., Zou Y., Yan Y. (2022). Fine-tuning gene expression for improved biosynthesis of natural products: from transcriptional to post-translational regulation. Biotechnol Adv.

[3] Engstrom M.D., Pfleger B.F. (2017). Transcription control engineering and applications in synthetic biology. Synthetic and Systems Biotechnology.

[4] Yim S.S., An S.J., Kang M., Lee J., Jeong K.J. (2013). Isolation of fully synthetic promoters for high-level gene expression in *Corynebacterium glutamicum*. Biotechnol Bioeng.

[5] Kosuri S., Goodman D.B., Cambray G., Mutalik V.K., Gao Y., Arkin A.P., Endy D., Church G.M. (2013). Composability of regulatory sequences controlling transcription and translation in *Escherichia coli*. Proc Natl Acad Sci USA.

[6] Zhang B., Zhou N., Liu Y.-M., Liu C., Lou C.-B., Jiang C.-Y., Liu S.-J. (2015). Ribosome binding site libraries and pathway modules for shikimic acid synthesis with *Corynebacterium glutamicum*. Microb Cell Factories.

[7] Mutalik V.K., Guimaraes J.C., Cambray G., Mai Q.A., Christoffersen M.J., Martin L., Yu A., Lam C., Rodriguez C., Bennett G., Keasling J.D., Endy D., Arkin A.P. (2013). Quantitative estimation of activity and quality for collections of functional genetic elements. Nat Methods.

[8] Salis H.M., Mirsky E.A., Voigt C.A. (2009). Automated design of synthetic ribosome binding sites to control protein expression. Nat Biotechnol.

[9] Zhang P., Wang H., Xu H., Wei L., Liu L., Hu Z., Wang X. (2023). Deep flanking sequence engineering for efficient promoter design using DeepSEED. Nat Commun.

[10] Huang Y.-K., Yu C.-H., Ng I.S. (2023). Precise strength prediction of endogenous promoters from *Escherichia coli* and J-series promoters by artificial intelligence. J Taiwan Inst Chem Eng.

[11] Wang Y., Wang H., Wei L., Li S., Liu L., Wang X. (2020). Synthetic promoter design in *Escherichia coli* based on a deep generative network. Nucleic Acids Res.

[12] Na D., Rbsdesigner Lee D. (2010). Software for designing synthetic ribosome binding sites that yields a desired level of protein expression. Bioinformatics.

[13] Jeschek M., Gerngross D., Panke S. (2016). Rationally reduced libraries for combinatorial pathway optimization minimizing experimental effort. Nat Commun.

[14] Swainston N., Dunstan M., Jervis A.J., Robinson C.J., Carbonell P., Williams A.R., Faulon J.L., Scrutton N.S., Kell D.B. (2018). PartsGenie: an integrated tool for optimizing and sharing synthetic biology parts. Bioinformatics.

[15] Schmitz A., Zhang F. (2021). Massively parallel gene expression variation measurement of a synonymous codon library. BMC Genom.

[16] Verma M., Choi J., Cottrell K.A., Lavagnino Z., Thomas E.N., Pavlovic-Djuranovic S., Szczesny P., Piston D.W., Zaher H.S., Puglisi J.D., Djuranovic S. (2019). A short translational ramp determines the efficiency of protein synthesis. Nat Commun.

[17] Kudla G., Murray A.W., Tollervey D., Plotkin J.B. (2009). Coding-sequence determinants of gene expression in *Escherichia coli*. Science.

[18] Mutalik V.K., Guimaraes J.C., Cambray G., Lam C., Christoffersen M.J., Mai Q.A., Tran A.B., Paull M., Keasling J.D., Arkin A.P., Endy D. (2013). Precise and reliable gene expression via standard transcription and translation initiation elements. Nat Methods.

[19] Jin E., Wong L., Jiao Y., Engel J., Holdridge B., Xu P. (2017). Rapid evolution of regulatory element libraries for tunable transcriptional and translational control of gene expression. Synthetic and Systems Biotechnology.

[20] Balakrishnan R., Mori M., Segota I., Zhang Z., Aebersold R., Ludwig C., Hwa T. (2022). Principles of gene regulation quantitatively connect DNA to RNA and proteins in bacteria. Science.

[21] Zrimec J., Börlin C.S., Buric F., Muhammad A.S., Chen R., Siewers V., Verendel V., Nielsen J., Töpel M., Zelezniak A. (2020). Deep learning suggests that gene expression is encoded in all parts of a co-evolving interacting gene regulatory structure. Nat Commun.

[22] Cheng T., Wang L., Sun C., Xie C. (2022). Optimizing the downstream MVA pathway using a combination optimization strategy to increase lycopene yield in *Escherichia coli*. Microb Cell Factories.

[23] Lee D.-H., Kim H., Sung B.-H., Cho B.K., Lee S.-G. (2023). Biofoundries: bridging automation and biomanufacturing in synthetic biology. Biotechnol Bioproc Eng.

[24] Wang Y., Cheng H., Liu Y., Liu Y., Wen X., Zhang K., Ni X., Gao N., Fan L., Zhang Z., Liu J., Chen J., Wang L., Guo Y., Zheng P., Wang M., Sun J., Ma Y. (2021). In-situ generation of large numbers of genetic combinations for metabolic reprogramming via CRISPR-guided base editing. Nat Commun.

[25] Wang Y., Liu Y., Liu J., Guo Y., Fan L., Ni X., Zheng X., Wang M., Zheng P., Sun J., Ma Y. (2018). MACBETH: multiplex automated *Corynebacterium glutamicum* base editing method. Metab Eng.

[26] Egbert R.G., Klavins E. (2012). Fine-tuning gene networks using simple sequence repeats. Proc Natl Acad Sci USA.

[27] Gibson D.G., Young L., Chuang R.Y., Venter J.C., Hutchison C.A., Smith H.O. (2009). Enzymatic assembly of DNA molecules up to several hundred kilobases. Nat Methods.

[28] Buccitelli C., Selbach M. (2020). mRNAs, proteins and the emerging principles of gene expression control. Nat Rev Genet.

[29] Chen C., Chen H., Zhang Y., Thomas H.R., Frank M.H., He Y., Xia R. (2020). TBtools: an integrative toolkit developed for interactive analyses of big biological data. Mol Plant.

[30] Valeri J.A., Soenksen L.R., Collins K.M., Ramesh P., Cai G., Powers R., Angenent-Mari N.M., Camacho D.M., Wong F., Lu T.K., Collins J.J. (2023). BioAutoMATED: an end-to-end automated machine learning tool for explanation and design of biological sequences. Cell Systems.

[31] Olson R.S., Moore J.H. (2016). Workshop on automatic machine learning.

[32] Byla E, Pang W: Deepswarm: optimising convolutional neural networks using swarm intelligence. In: Advances in computational intelligence systems: contributions presented at the 19th UK workshop on computational intelligence, september 4-6, 2019, Portsmouth, UK 19: 2020. Springer: 119-130.

[33] Jin H., Song Q., Hu X. (2019). Proceedings of the 25th ACM SIGKDD international conference on knowledge discovery & data mining.

[34] Chen T., C G. (2016). XGBoost: a scalable tree boosting system. Proceedings of the 22nd ACM SIGKDD International Conference on Knowledge Discovery and Data Mining.

[35] Friedman J.H. (2001). Greedy function approximation: a gradient boosting machine. Ann Stat.

[36] Feurer M., Eggensperger K., Falkner S., Lindauer M., Hutter F. (2022). Auto-sklearn 2.0: hands-free automl via meta-learning. J Mach Learn Res.

[37] Wang M., Shi Z., Gao N., Zhou Y., Ni X., Chen J., Liu J., Zhou W., Guo X., Xin B., Shen Y., Wang Y., Zheng P., Sun J. (2023). Sustainable and high-level microbial production of plant hemoglobin in *Corynebacterium glutamicum*. Biotechnology for Biofuels and Bioproducts.

[38] Liu J., Liu M., Shi T., Sun G., Gao N., Zhao X., Guo X., Ni X., Yuan Q., Feng J., Liu Z., Guo Y., Chen J., Wang Y., Zheng P., Sun J. (2022). CRISPR-assisted rational flux-tuning and arrayed CRISPRi screening of an L-proline exporter for L-proline hyperproduction. Nat Commun.

[39] Wang J., Xue N., Pan W., Tu R., Li S., Zhang Y., Mao Y., Liu Y., Cheng H., Guo Y., Yuan W., Ni X., Wang M. (2023). Repurposing conformational changes in ANL superfamily enzymes to rapidly generate biosensors for organic and amino acids. Nat Commun.

[40] Wang Y., Li S., Xue N., Wang L., Zhang X., Zhao L., Guo Y., Zhang Y., Wang M. (2023). Modulating sensitivity of an erythromycin biosensor for precise high-throughput screening of strains with different characteristics. ACS Synth Biol.

[41] Rudd K.E. (2000). EcoGene: a genome sequence database for *Escherichia coli* K-12. Nucleic Acids Res.

[42] Wen X., Zhang Y., Cheng H., An J., Guo Y., Wang L., Wang M. (2021). A CRISPR/dCas9-assisted system to clone toxic genes in *Escherichia coli*. Biochim Biophys Acta Gen Subj.

[43] El Qaidi S., Allemand F., Oberto J., Plumbridge J. (2009). Repression of *galP*, the galactose transporter in *Escherichia coli*, requires the specific regulator of N-acetylglucosamine metabolism. Mol Microbiol.

[44] Boël G., Letso R., Neely H., Price W.N., Wong K.H., Su M., Luff J., Valecha M., Everett J.K., Acton T.B., Xiao R., Montelione G.T., Aalberts D.P., Hunt J.F. (2016). Codon influence on protein expression in *E. coli* correlates with mRNA levels. Nature.

[45] Goodman D.B., Church G.M., Kosuri S. (2013). Causes and effects of N-terminal codon bias in bacterial genes. Science.

[46] Hossain A., Lopez E., Halper S.M., Cetnar D.P., Reis A.C., Strickland D., Klavins E., Salis H.M. (2020). Automated design of thousands of nonrepetitive parts for engineering stable genetic systems. Nat Biotechnol.

[47] Wu C.C., Peterson A., Zinshteyn B., Regot S., Green R. (2020). Ribosome collisions trigger general stress responses to regulate cell fate. Cell.

