# Supplementary material for: Automated characterization and analysis of expression compatibility between regulatory sequences and metabolic genes in Escherichia coli
Source: Synth Syst Biotechnol. 2024 May 17;9(4):647–57. doi: 10.1016/j.synbio.2024.05.010 (PMC11137365; doi:10.1016/j.synbio.2024.05.010)
Supplement: Multimedia component 2 [file mmc2.docx]

Automated characterization and analysis of expression compatibility between regulatory sequences and metabolic genes in *Escherichia coli*

Xiao Wen^1,2,3^, Jiawei Lin^2,4^, Chunhe Yang^2,4^, Ying Li^2,4^, Haijiao Cheng^2,3^, Ye Liu^2,3^, Yue Zhang^2,3^, Hongwu Ma^2,3^, Yufeng Mao^2,3,*^, Xiaoping Liao^2,3,*^, Meng Wang^1,2,3,*^

^1^ School of Life Sciences, Division of Life Sciences and Medicine, University of Science and Technology of China, Hefei 230026, China.

^2^ Tianjin Institute of Industrial Biotechnology, Chinese Academy of Sciences, Tianjin 300308, China.

^3^ Key Laboratory of Engineering Biology for Low-Carbon Manufacturing, Tianjin 300308, China.

^4^ School of Biological Engineering, Tianjin University of Science and Technology, Tianjin 300457, China.

*Corresponding authors:

Dr. Yufeng Mao, E-mail: maoyf@tib.cas.cn

Dr. Xiaoping Liao, E-mail: liao_xp@tib.cas.cn

Dr. Meng Wang, E-mail: wangmeng@tib.cas.cn


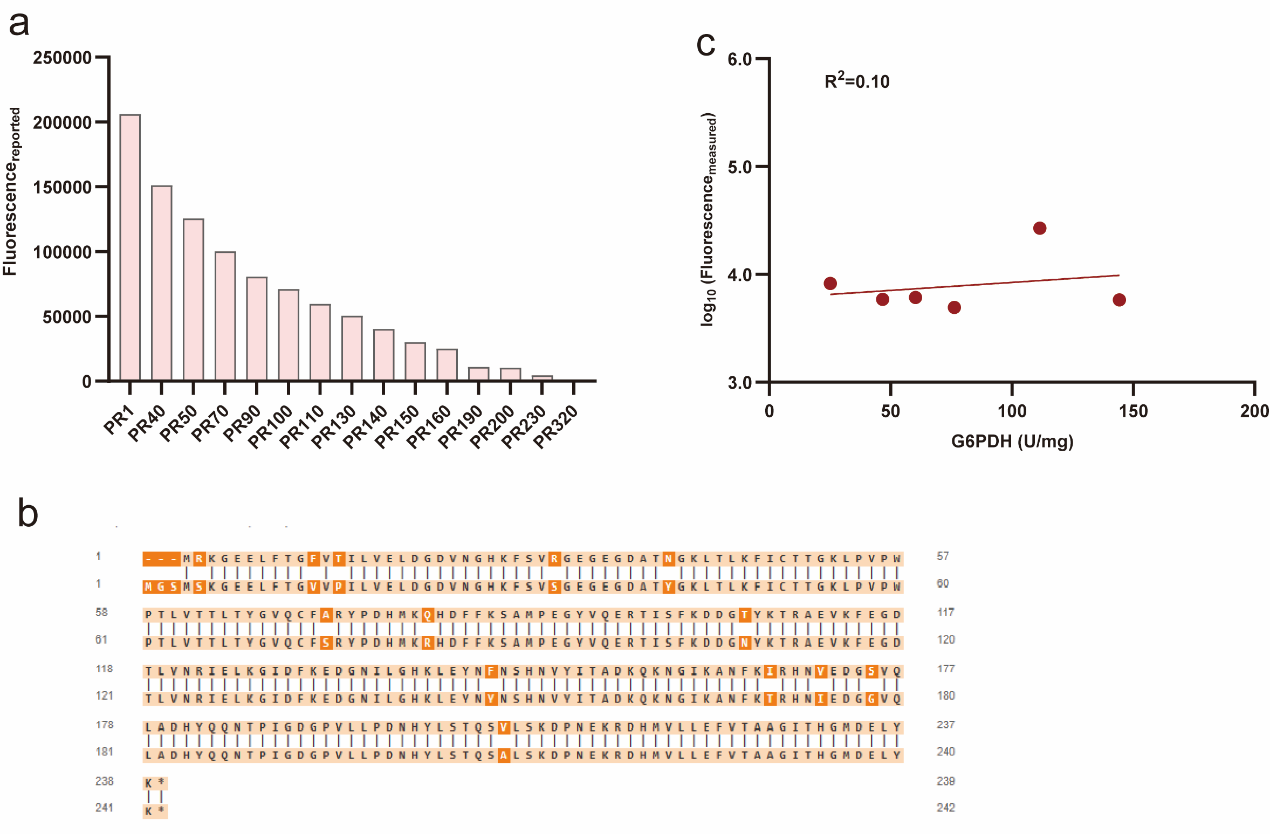


Supplementary Fig. 1. Evaluation of expression compatibility using different fluorescent proteins. a, The fluorescence levels of 15 regulatory sequences provided in the literature^[1]^. b, The sequence alignment results of two fluorescent proteins (GFP and sfGFP). c, Correlation analysis of measured fluorescence values with G6PDH enzyme activities.


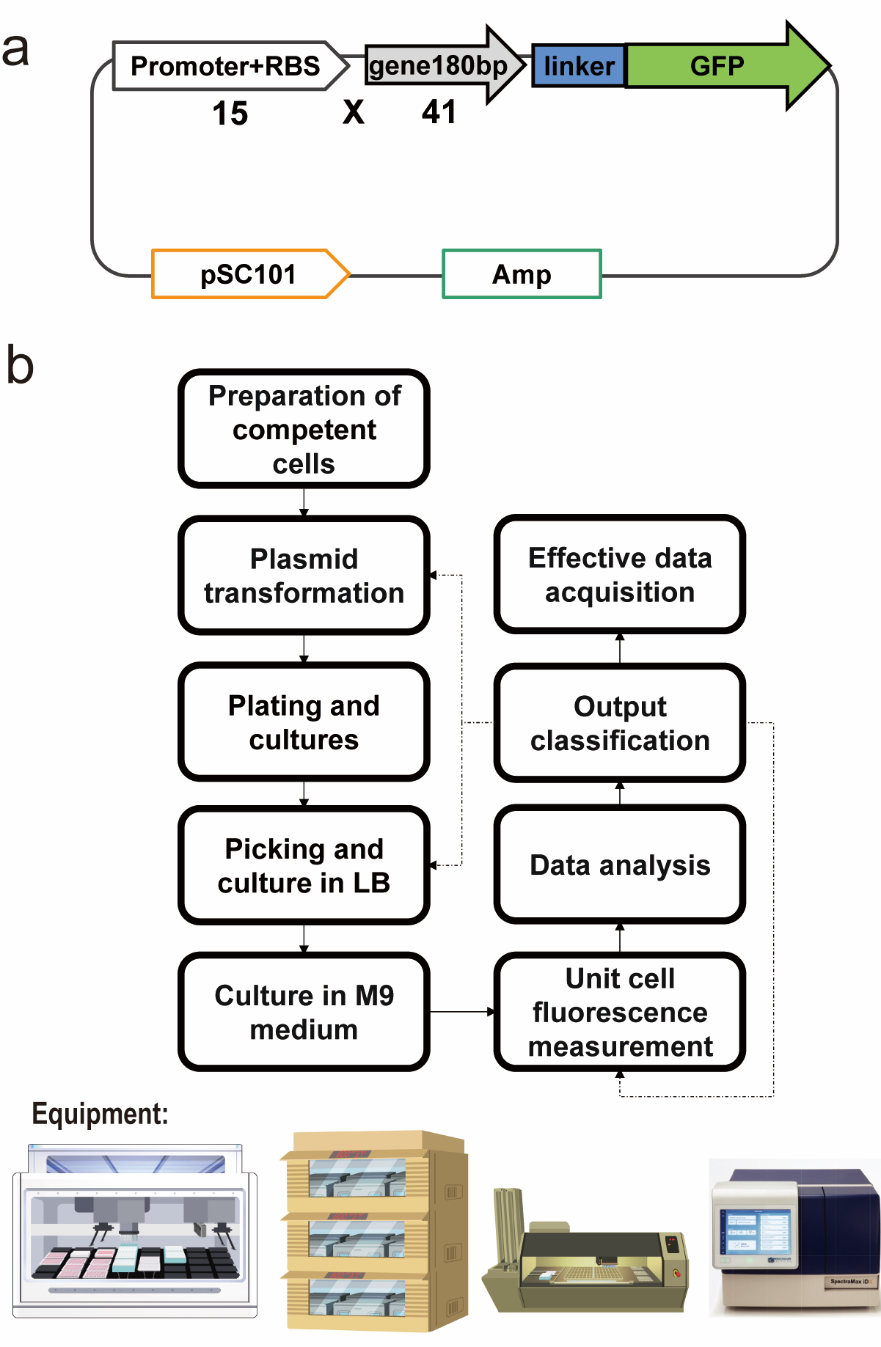


Supplementary Fig. 2. High-throughput automated characterization workflow for the fluorescence levels of fused protein. a, Schematic diagram of plasmids for the combinations of fused GOI_180_-linker-*gfp* genes and regulatory sequences. b, Workflow of automated regulatory sequence characterization.


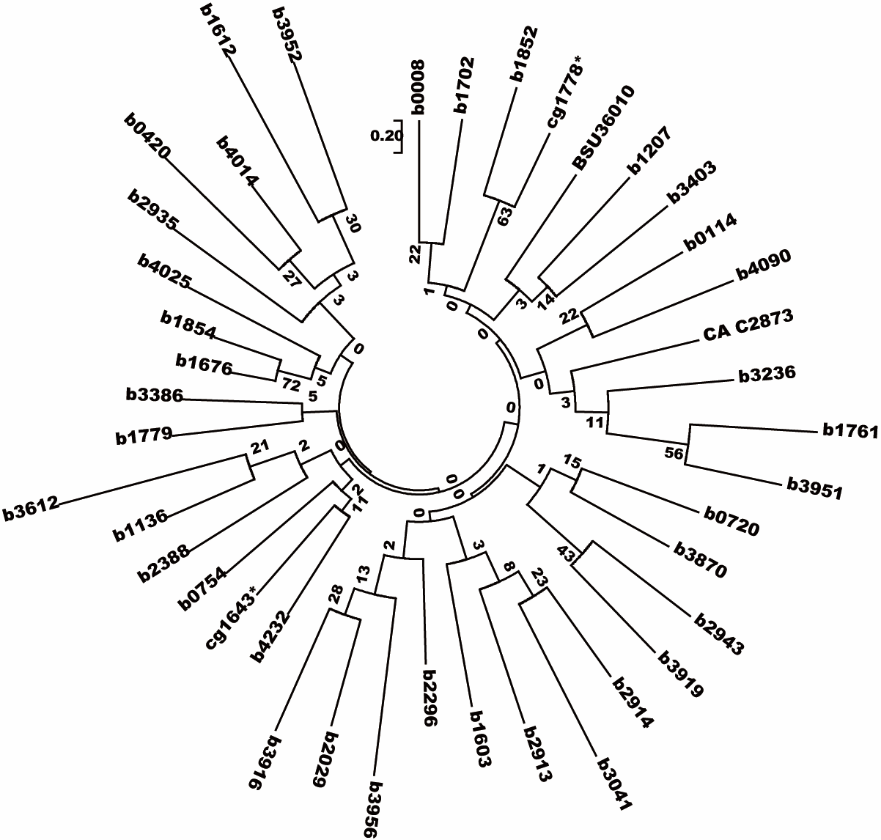


Supplementary Fig. 3. Phylogenetic tree of the alignment of the initial 180 bp nucleotide sequences for the CDS of all genes.


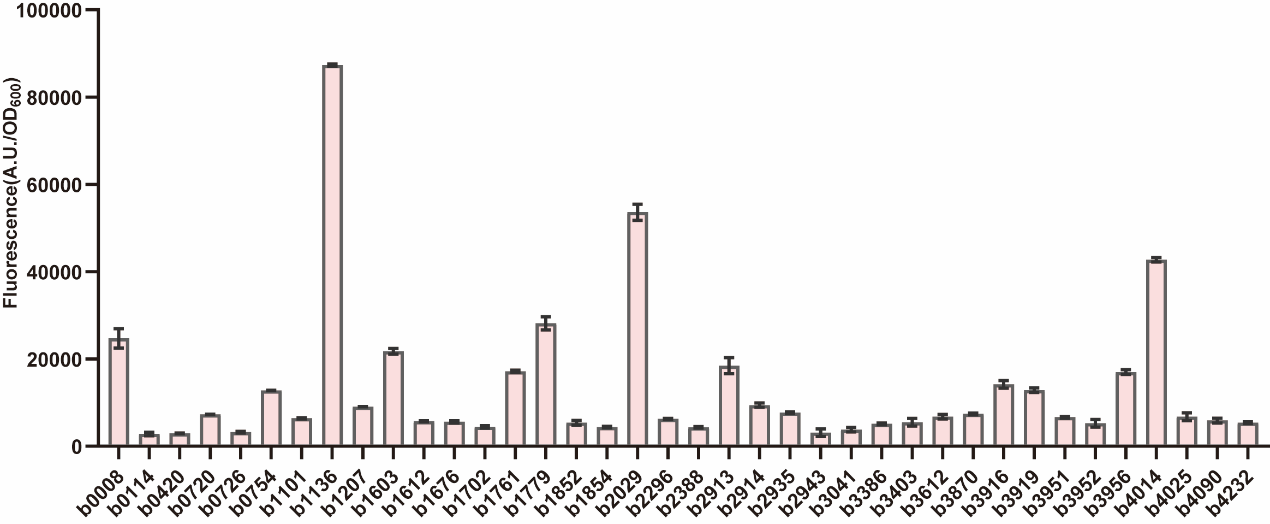


Supplementary Fig. 4. Fluorescence intensity of GFP fusions based on 36 endogenous genes under their natural regulatory sequences.


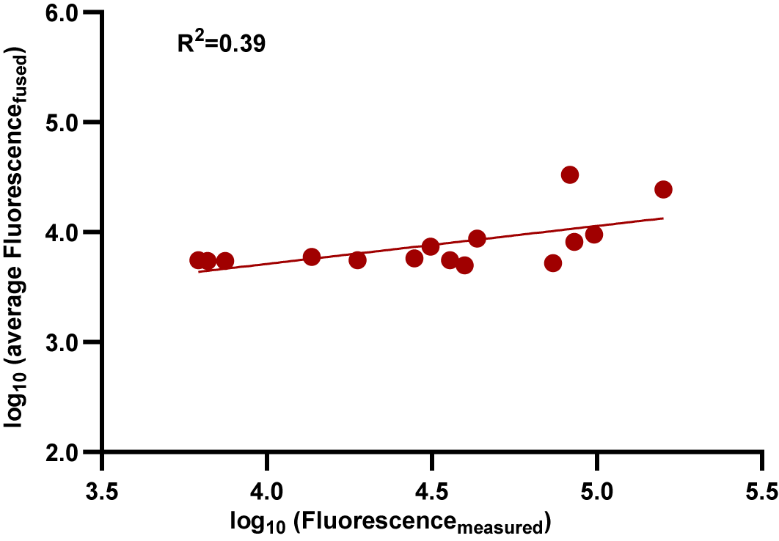


Supplementary Fig. 5. Correlation analysis of the average fluorescence intensity of fusion proteins based on different metabolic genes with measured fluorescence from the *gfp* gene under the same set of regulatory sequences.


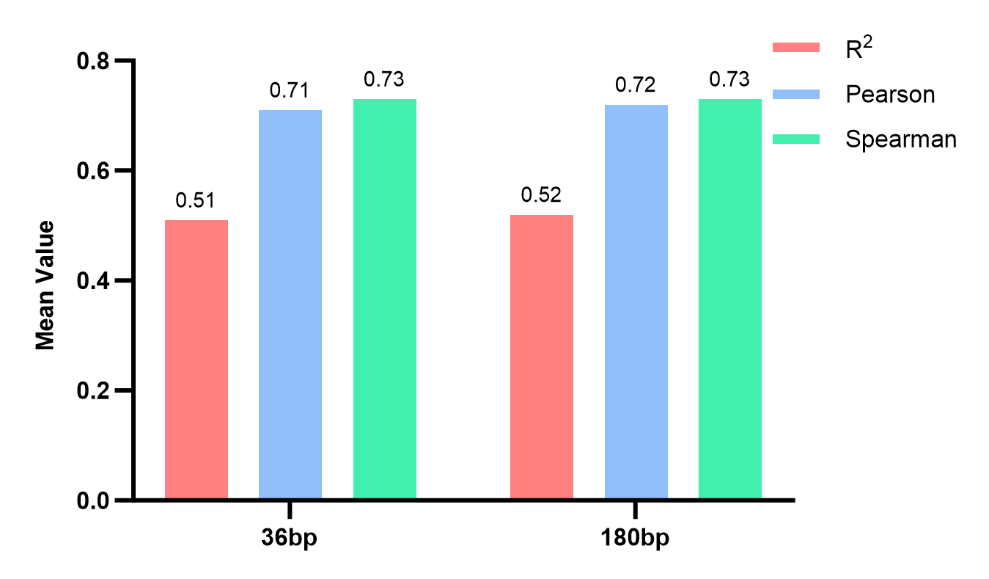


Supplementary Fig. 6. Comparison of prediction results from the combination of promoter, RBS and different truncated CDS (36 bp versus 180 bp) based on DeepSwarm architecture.

[1] KOSURI S, GOODMAN D B, CAMBRAY G, et al. Composability of regulatory sequences controlling transcription and translation in *Escherichia coli* [J]. Proceedings of the National Academy of Sciences, 2013, 110(34): 14024-9.
